# Remote ischemic conditioning in septic shock (RECO-Sepsis): study protocol for a randomized controlled trial

**DOI:** 10.1186/s13063-019-3406-4

**Published:** 2019-05-22

**Authors:** Martin Cour, Marielle Buisson, Kada Klouche, Radhia Bouzgarrou, Carole Schwebel, Jean-Pierre Quenot, Fabrice Zeni, Pascal Beuret, Michel Ovize, Laurent Argaud

**Affiliations:** 1 Hospices Civils de Lyon, Hôpital Edouard Herriot, Service de Médecine Intensive-Réanimation, 5, place d’Arsonval, 69437 Lyon Cedex 03, France; 2 Faculté de Médecine Lyon-Est, Université de Lyon, Université Claude Bernard Lyon 1, Lyon, France; 3 Centre d’investigation Clinique – INSERM 1407, Hospices Civils de Lyon, Hôpital Cardiologique Louis Pradel, Bron, France; 4 Service de Réanimation Médicale, CHU de Montpellier, Montpellier, France; 5 Service de Réanimation Médicale, CHU Gabriel Montpied, Clermont-Ferrand, France; 6 Service de Réanimation Médicale, CHU Albert Michallon, Grenoble, France; 7 Service de Réanimation Médicale, CHU François-Mitterrand, Dijon, France; 8 Service de Réanimation Médicale, CHU de Saint-Etienne, Saint-Etienne, France; 9 Service de Réanimation polyvalente, CHR de Roanne, Roanne, France

**Keywords:** Septic shock, Intensive care, Multiple organ failure, Sequential Organ Failure Assessment (SOFA) score, Remote ischemic conditioning, Ischemia-reperfusion, Reperfusion injury

## Abstract

**Background:**

Septic shock is a major public health problem that is associated with up to 50% mortality. Unfavorable outcomes are mainly attributed to multiple organ failure (MOF) resulting from an uncontrolled inflammatory response and ischemia-reperfusion processes. REmote ischemic COnditioning (RECO) is a promising intervention to prevent ischemia-reperfusion injury. We hypothesize that RECO would reduce the severity of septic shock-induced MOF.

**Methods/design:**

RECO in septic shock patients (RECO-Sepsis study) is an ongoing, prospective, multicenter, randomized, open-label trial, testing whether RECO, as an adjuvant therapy to conventional treatment in septic shock, decreases the severity of MOF as assessed by the Sequential Organ Failure Assessment (SOFA) score. Adult patients admitted to an intensive care unit with documented or suspected infection, lactatemia > 2 mmol/l, and treated with norepinephrine for less than 12 h are potentially eligible for the study. Non-inclusion criteria are: having expressed the wish not to be resuscitated, contraindication for the use of a brachial cuff on both arms, intercurrent disease with an expected life expectancy of less than 24 h, cardiac arrest, and pregnant or breastfeeding women. After enrollment, patients are randomized (*n* = 180) 1:1 to receive RECO or no adjunctive intervention. RECO consists of four cycles of cuff inflation to 200 mmHg for 5 min and then deflation to 0 mmHg for another 5 min. RECO is performed at inclusion and repeated 12 and 24 h later. The primary endpoint is the mean daily SOFA score up to day 4 after inclusion. Secondary outcomes include the need for organ support, hospital length of stay, and 90-day mortality.

**Discussion:**

Results of this proof-of-concept trial should provide information on the efficacy of RECO in patients with septic shock.

**Trial registration:**

ClinicalTrials.gov, ID: identifier: NCT03201575. Registered on 28 June 2017.

**Electronic supplementary material:**

The online version of this article (10.1186/s13063-019-3406-4) contains supplementary material, which is available to authorized users.

## Background

Septic shock is a major and growing public health problem affecting millions of patients worldwide annually and 10 to 30% of patients admitted to intensive care units (ICUs) [1]. Despite regularly updated international guidelines and significant improvement in the treatment of septic shock, short-term mortality, which is mostly attributed to multiple organ failure (MOF), still remains as high as 50% [1, 2]. In view of these alarming figures, there is a compelling need for new therapeutic approaches to prevent MOF and thereby improve survival in this disease.

The pathophysiology of septic shock-induced MOF is complex and remains incompletely understood. It is recognized that it results, at least in part, from an uncontrolled systemic inflammatory response and ischemia/reperfusion (I/R) processes related to transient arterial hypotension, low cardiac output, and compromised microcirculation with cellular hypoxia/reoxygenation and mitochondrial dysfunction [2–4]. However, among the first-line treatments recommended for patients with septic shock (i.e., antibiotics, source control of infection, fluids, and vasoactive drugs), none specifically targets I/R injury [2].

REmote ischemic COnditioning (RECO) represents an innovative strategy to protect organs (e.g., brain, heart, kidneys) against the deleterious effect of I/R through activation of cell survival pathways and modulation of the inflammatory response [5–8]. RECO usually consists of applying brief and repeated cycles of non-lethal ischemia alternating with reperfusion by inflating and deflating a blood pressure cuff placed around a limb [5–8]. Over the past few years, this simple, inexpensive, non-invasive, and innocuous intervention has been investigated in numerous clinical trials with promising results [9–13]. For instance, it has been shown that RECO may limit myocardial damage after both acute myocardial infarction [9] and on-pump cardiac surgery [10]. Beyond protection of the heart, RECO has also been reported to be nephroprotective and neuroprotective, suggesting a ubiquitous effect of this intervention in humans [11–13]. Specifically for septic shock, only pre-clinical data are available and these indicate clear benefits of RECO, including survival, in both small and large animals [14–16].

To the best of our knowledge, RECO has not been tested in patients with septic shock. We have, therefore, implemented a multicenter, randomized controlled trial (RECO-Sepsis study) to investigate whether ischemic conditioning applied by inflating/deflating a cuff around an upper limb would limit the severity of MOF in septic shock patients.

## Methods/design

### Design and setting

**Fig. 1:**
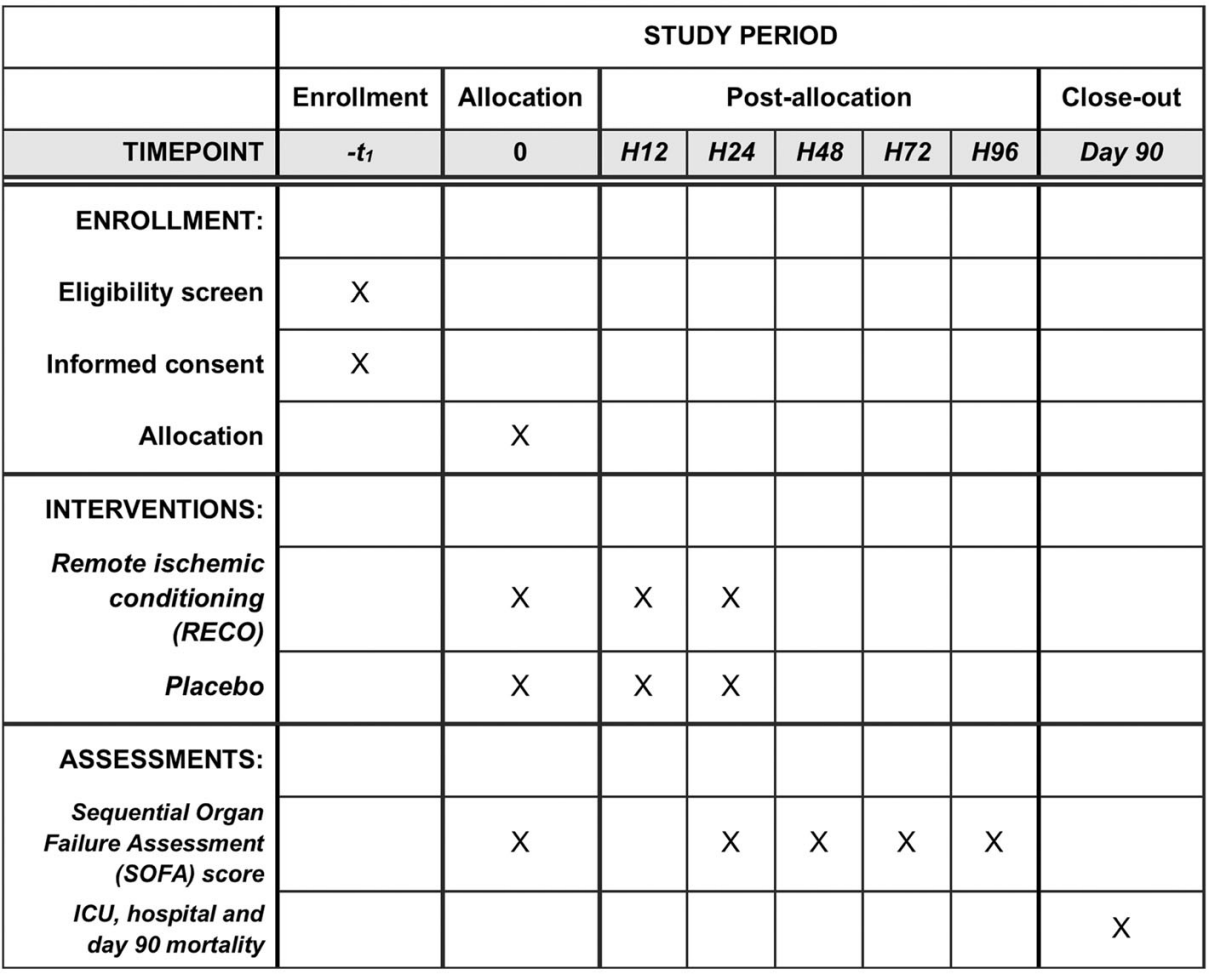
Schedule of enrollment, interventions, and assessments according to Standard Protocol Items: Recommendations for Interventional Trials (SPIRIT). *ICU* intensive care unit

**Fig. 2:**
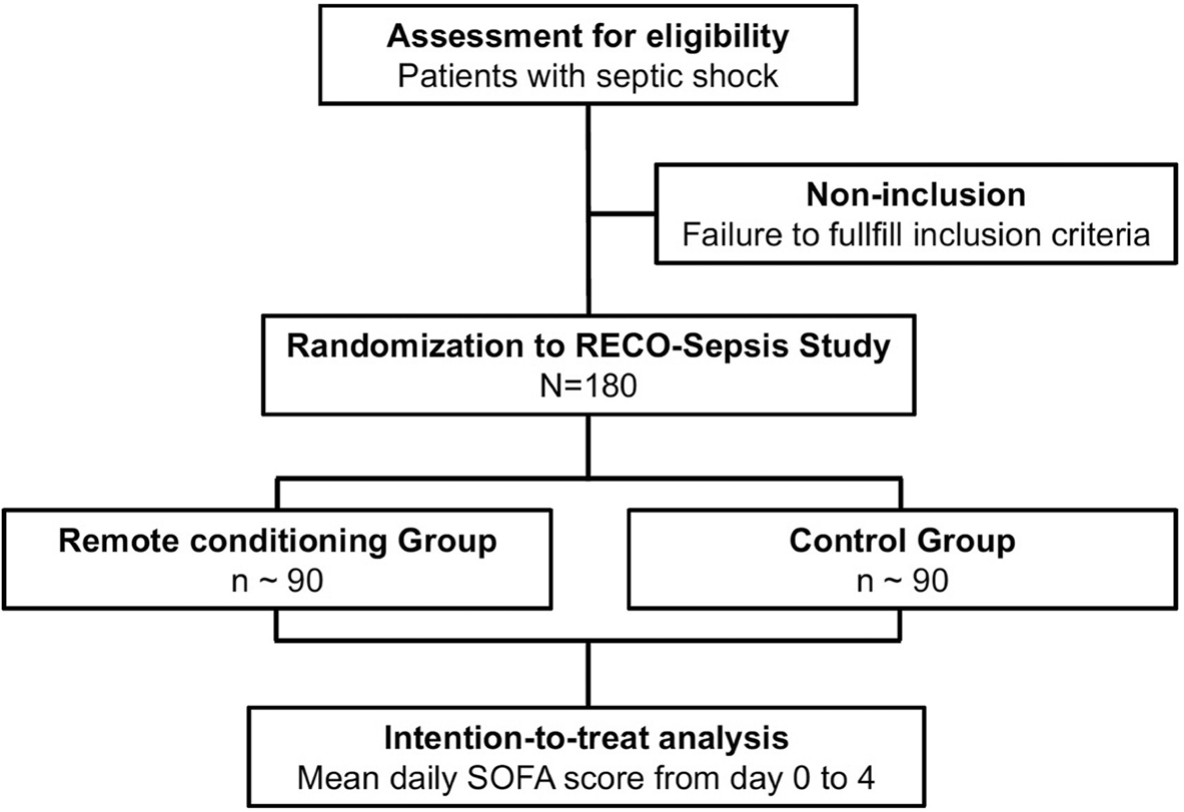
Flow chart

The trial protocol was developed in accordance with the SPIRIT (Standard Protocol Items: Recommendations for Interventional Trials) guidelines [17] (Additional file 1). RECO-Sepsis is a prospective, multicenter, randomized, open-label, parallel-group trial, testing whether RECO as an adjuvant therapy to conventional treatment in septic shock would decrease the severity of MOF as measured by the Sequential Organ Failure Assessment (SOFA) score [18–21]. The trial is conducted in French intensive care units (ICUs). An overview of enrollment, interventions, and follow-up of participants in the RECO-Sepsis trial is shown in Figs. 1 and 2. The trial is supported by a grant from the French Ministry of Health (Programme Hospitalier de Recherche Clinique Inter-régional 2016) and the study sponsor is the Hospices Civils de Lyon. The study was registered at ClinicalTrials.gov (NCT03201575) on 28 June 2017.

### Ethics

The trial is conducted in accordance with the principles of the Declaration of Helsinki 1964 as revised in 2013 and the International Conference of Harmonization Guidelines for Good Clinical Practice (ICH GCP E6 guidelines). In accordance with French laws, the trial was authorized by the French national drug agency (Agence Nationale de la Sécurité du Médicament, ANSM) on 23 August 2017. The study was also approved by the Institutional Review Board (Comité de Protection des Personnes CCP Ile-de-France X) on 5 September 2017 (n°2017-A01684–49).

### Participants

All patients admitted to the participating centers are screened against eligibility criteria. Inclusion criteria are as follows: adult patients (aged 18 years or older); hospitalized in ICU for less than 24 h, and septic shock evolving for less than 12 h. Septic shock is defined according to the 2016 revised standard consensus criteria: documented or suspected infection, lactatemia > 2 mmol/l, and norepinephrine administration to maintain a mean arterial pressure (MAP) above 65 mmHg after adequate fluid resuscitation, [22]. Non-inclusion criteria are as follows: having expressed the wish not to be resuscitated, contraindication for the use of a brachial cuff on both arms, intercurrent disease with an expected life expectancy of less than 24 h, cardiac arrest, pregnant or breastfeeding women, previous inclusion in the study or participation in another interventional study*,* lack of French national health insurance coverage, and being under judicial protection.

### Informed consent

Informed consent that details the study design and outcomes must be obtained prior to inclusion. If the patient is able to consent but cannot write, an oral consent can be obtained in the presence of an impartial witness, independent of the study investigator. It is expected that, in most cases, it will not be possible to obtain prospective consent from the patient due to incapacity resulting from critical illness and its treatment. Under such conditions, written consent is sought from next of kin. All surviving patients are informed about the trial once they regain capacity and are asked to give consent to their ongoing involvement in the trial. Patient or their relatives can withdraw their consent and discontinue the participation in the study at any time upon request.

### Randomization

After consent, eligible patients are randomly assigned to received RECO or not. Randomization is stratified by center on a 1:1 basis in permuted blocks. Randomization is performed online using the ClinSight™ software (Ennov Clinical Software, Paris, France).

### Intervention

As soon as possible after randomization to either the RECO group or the Control group, a standard CE-approved blood pressure cuff (Tensiomètre Manopoire Duo Colson®, Drive DeVilbiss Healthcare, Frouard, France) is placed, deflated, around one of the patient’s upper arms. In line with the recommendations for non-invasive measurement of blood pressure, the size of the cuff is chosen to accommodate the patient’s arm [23]. In the RECO group, ischemic conditioning consists of four cycles of cuff inflation to 200 mmHg for 5 min and then deflation to 0 mmHg for another 5 min (40 min total duration of the intervention). Both the safety and the efficacy of this standard protocol has been demonstrated in previous clinical trials [5–7, 9–13]. In the Control group, the cuff is left in place, deflated, for 40 min (sham procedure). The RECO or the sham procedure is repeated at 12 and 24 h after inclusion. Standard operation procedures (SOP) describing the interventions are available for all investigators. A written checklist is completed by the investigator performing the intervention to ensure protocol adherence. With the exception of the tested intervention, patients receive standard care in accordance with international guidelines [2].

### Primary outcome

The primary outcome of this trial is the severity of sepsis-induced MOF as assessed by the mean daily SOFA score from inclusion (day 0) to the fourth day after inclusion (day 4) or to the day of death if it occurs before day 4. The SOFA score is an objective tool to quantitatively describe the degree of organ dysfunction over time [18–21]. The mean daily SOFA score, which closely correlates with mortality in critically ill patients [19], is regularly used as a surrogate outcome for death in sepsis trials [24, 25]. The choice of a mean SOFA score helps to solve the “truncated by death” issue as all patients contribute scores and no imputation of data is needed if a patient dies. As shown in Table 1, the SOFA score ranges from 0 to 24 (higher scores indicate more severe organ failure), with 0 to 4 points assigned for each of six organ dysfunctions (i.e., respiratory, coagulation, liver, cardiovascular, central nervous system, and renal). As previously described, a daily SOFA score is calculated using the most abnormal clinical and/or biological values in the previous 24 h (or from the start of the considered 24-h period to death) [18–21]. Predefined rules for dealing with potential missing data have been established.

**Table 1:**
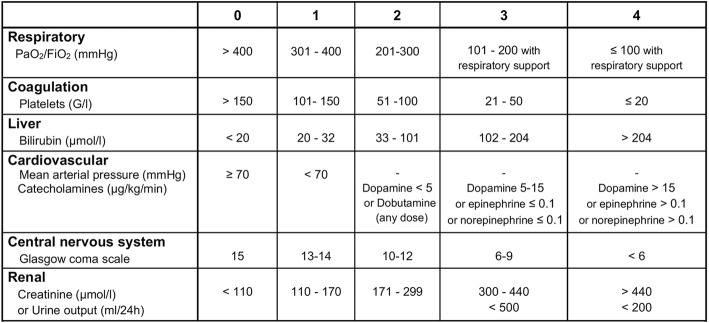
Sequential Organ Failure Assessment (SOFA) score. *PaO*_*2*_ partial pressure of oxygen, *FiO*_*2*_ Fraction of inspired oxygen

### Secondary outcomes

The main secondary endpoints include: mean SOFA score from day 0 to day 4 after exclusion of the neurological subscore; daily SOFA scores from day 0 to day 4; daily SOFA subscores for each organ/system from day 0 to day 4; variation of SOFA scores from day 0 to day 4; number of days alive without organ support (i.e., invasive mechanical ventilation, renal replacement therapy, or vasoactive drugs) on day 28; length of ICU and hospital stay; all-cause mortality in ICU, hospital, on day 28 and on day 90.

### Data collection

The following data are recorded: demographics, concurrent medical conditions and comorbidities, sepsis diagnosis and treatment, severity of illness and organ dysfunction scores, vital signs and laboratory results, characteristics of the RECO procedure, life-sustaining therapies, and outcomes (follow-up: 90 days). Data are entered into a web-based electronic case report form (eCRF, ClinSight™ software) from patient notes (source) under the supervision of the trial site investigators. A unique numeric identifier is assigned to each patient. All the data entered in the study database are anonymized in order to ensure confidentiality. Assessments are made at inclusion, and on days 1, 2, 3, 4, 28, and 90. From the eCRFs, the trial database will be established. According to French laws, all original records (including informed consent forms, eCRF, and relevant correspondences) will be archived at trial sites for 25 years.

### Safety

Given that the trial is being conducted in critically ill patients, a significant number of patients are likely to experience adverse events (AEs) not related to the intervention. Therefore, only grade-3 or more AEs are reported. The following clinical outcomes from sepsis will not be recorded as AEs unless the local investigator deems the event to be related to the study: cardiovascular failure, respiratory failure, hepatic failure, renal failure, hematological failure, or neurological failure. While no significant RECO-related AE is expected based on previously published studies, all mechanical complications related to the use of the blood pressure cuff and leading to a medical/surgical intervention are systematically reported as serious AEs to the sponsor center within 24 h and to the relevant authorities in accordance with current French regulations. Any death occurring within 90 days after inclusion is also reported as a serious AE. All AEs are assessed for relationship with the tested intervention. No data safety monitoring board will be ask to monitor the study.

### Monitoring

Monitoring on site of all centers is performed by the study sponsor. The monitor will review the entries into the eCRF on the basis of source documents. Monitors will review the consent forms and all data contributing to the primary outcome. The investigators must give access to all essential source data and must provide support at all times to the monitor. The monitor also ensures regularly that the trial is conducted according to the protocol and regulatory requirements. In each center, a monitoring is planned after the enrollment of the first two patients and afterwards after every six patients.

### Study organization

The RECO-Sepsis study is coordinated by the Clinical Investigation Center (CIC) located in Lyon, France. Each participating center has a senior investigator as principal investigator with strong clinical trial experience. All trial-related processes follow the SOP of the RECO-Sepsis study protocol. The RECO-Sepsis Steering Committee is co-chaired by Martin Cour, MD, PhD and Laurent Argaud, MD, PhD (Claude Bernard University of Lyon 1 – Edouard Herriot Hospital, Lyon, France). The Steering Committee is responsible for the scientific content of the protocol and oversees the trial operations. The Steering Committee will have full access to all the data in the study and will have final responsibility for the decision to submit the results for publication.

### Role of the funding source

The funding source has no role in the study design, data collection, data analysis, data interpretation and writing of the final report.

### Sample size

No a priori evidence suggests the magnitude of the effect of RECO on the severity of MOF in septic shock patients. Under the alternative hypothesis of an expected difference of half the standard deviation (effect size: 0.5) of the mean daily SOFA score of the first 4 days after inclusion, at least 168 patients have to be enrolled to reject the null hypothesis of a similar mean SOFA score in both arms with a power of 90% (β = 10%) and type 1 error at α = 5% (two-tailed). Even though there is no published data regarding the mean daily SOFA score in patients meeting our eligibility criteria, one can assume a mean value ranging from 8 to 12 with a standard deviation ranging from 4 to 6. Thus, for example, if the standard deviation is found to be 4, then a decrease of 2 or more in the mean daily SOFA score will be needed to reach significance. We planned to recruit an additional 7% patients (*n* = 12) to account for potential loss to follow-up and withdrawal of consent. Thus, the planned sample size is 180 patients (90 patients in each arm). Based on the enrollment capacity of the participating centers, the recruitment is expected to be completed within 18 months. The sample size was calculated with the use of nQuery Advisor, version 5.0 (Statistical solution, Cork, Ireland).

### Statistical analysis

The trial will end once 180 patients have been enrolled and have completed the 90 days’ follow-up. No interim analysis will be planned for this study. A full statistical analysis plan will be drawn up by the CIC (Lyon) before the trial database is locked. Prior to performing any statistical tests or fitting statistical models, an exploratory analysis of the baseline variables will be completed. The number and pattern of missing data for baseline variables and outcomes will be established by forming appropriate tables and likely causes of any missing values will be investigated. Variables will be expressed as median and interquartile range (IQR) or number and proportion, as appropriate. Statistical analyses of the primary and secondary efficacy endpoints will primarily be based on the intent-to-treat population. The main analysis of the primary endpoint, the mean daily SOFA score from day 0 to day 4, will be performed by fitting a mixed-effects linear model with a fixed effect for the assigned treatment and a random centre effect. Secondary outcomes will be analyzed with appropriate statistical methods including the Wilcoxon rank sum, chi^2^ and log-rank tests. The same analysis will be performed on the per-protocol population, which will include all randomized patients who have received the intervention in accordance with the allocated group without any major protocol deviation. A probability value of < 0.05 will be considered to indicate statistical significance and 95% confidence intervals will be calculated.

### Dissemination of the results

The final trial report will be submitted to high-quality, peer-reviewed journals regardless of the results. The results of the trial will be reported in accordance with the Consolidated Standards of Reporting Trials (CONSORT) Statement [26, 27]. The Steering Committee will grant authorship depending on personal input according to the Vancouver definitions. All trial sites with at least one randomized patient will be granted at least one authorship. All trial sites and trial site investigators will be acknowledged. Side studies will be allowed if supported by the Steering Committee.

## Discussion

The protocol describes the first randomized controlled trial designed to evaluate the efficacy of RECO in septic shock patients. Conduct of the RECO-Sepsis trial is in broad agreement with the international guidelines. The RECO-Sepsis trial has sufficient power to detect a significant decrease in the severity of septic shock-induced MOF, which is known to be strongly correlated with outcomes. We empirically chose to repeat three times the RECO procedure within the first 24 h after randomization to enhance the potential effects of the intervention. We acknowledge that, in absence of comparison data on different modalities of RECO in septic shock, we cannot be certain that our procedure is the most protective in this field. The results of this proof-of-concept trial will not provide a definitive answer on whether RECO is useful to improve long-term survival in septic shock, but are expected to provide essential data for the design of trials with a hard endpoint.

## Trial status

The RECO-Sepis trial is currently recruiting patients. Inclusion began on 15 November 2017. The estimated inclusion period is 18 months.

## Additional file

Additional file 1: Standard Protocol Items: Recommendations for Interventional Trials (SPIRIT) 2013 Checklist: recommended items to address in a clinical trial protocol and related documents. (DOC 124 kb)

## Abbreviations

AE: Adverse event
CONSORT: Consolidated Standards of Reporting Trials
eCRF: Electronic case report form
I/R: Ischemia/reperfusion
ICU: Intensive care unit
MAP: Mean arterial pressure
MOF: Multiple organ failure
RECO: REmote ischemic COnditioning
SOFA: Sequential Organ Failure Assessment
SOP: Standard operating procedure
SPIRIT: Standard Protocol Items: Recommendations for Interventional Trials

## References

[1] Pavon A, Binquet C, Kara F, Martinet O, Ganster F, Navellou JC (2013). Profile of the risk of death after septic shock in the present era: an epidemiologic study. Crit Care Med.

[2] Rhodes A, Evans LE, Alhazzani W, Levy MM, Antonelli M, Ferrer R (2017). Surviving Sepsis Campaign: International guidelines for management of sepsis and septic shock: 2016. Intensive Care Med.

[3] Hotchkiss RS, Moldawer LL, Opal SM, Reinhart K, Turnbull IR, Vincent JL (2016). Sepsis and septic shock. Nat Rev Dis Primers.

[4] Ait-Oufella H, Bourcier S, Lehoux S, Guidet B (2015). Microcirculatory disorders during septic shock. Curr Opin Crit Care.

[5] Cour M, Gomez L, Mewton N, Ovize M, Argaud L (2011). Post-conditioning: from the bench to bedside. J Cardiovasc Pharmacol Ther.

[6] Ovize M, Thibault H, Prizylenk K (2013). Myocardial conditioning: opportunities for clinical translation. Circ Res.

[7] Hausenloy DJ, Yellon DM (2016). Ischaemic conditioning and reperfusion injury. Nat Rev Cardiol.

[8] Hadebe N, Cour M, Lecour S (2018). The SAFE pathway for cardioprotection: is it a promising target?. Basic Res Cardiol.

[9] Bøtker HE, Kharbanda R, Schmidt MR, Bøttcher M, Kaltoft AK, Terkelsen CJ (2010). Remote ischaemic conditioning before hospital admission, as a complement to angioplasty, and effect on myocardial salvage in patients with acute myocardial infarction: a randomised trial. Lancet..

[10] Thielmann M, Kottenberg E, Kleinbongard P, Wendt D, Gedik N, Pasa S (2013). Cardioprotective and prognostic effects of remote ischaemic preconditioning in patients undergoing coronary artery bypass surgery: a single-centre randomised, double-blind, controlled trial. Lancet..

[11] Er F, Nia AM, Dopp H, Hellmich M, Dahlem KM, Caglayan E (2012). Ischemic preconditioning for prevention of contrast medium-induced nephropathy: randomized pilot RenPro Trial (Renal Protection Trial). Circulation..

[12] Zarbock A, Schmidt C, Van Aken H, Wempe C, Martens S, Zahn PK (2015). Effect of remote ischemic preconditioning on kidney injury among high-risk patients undergoing cardiac surgery: a randomized clinical trial. JAMA..

[13] England TJ, Hedstrom A, O’Sullivan S, Donnelly R, Barrett DA, Sarmad S (2017). RECAST (Remote Ischemic Conditioning After Stroke Trial): a pilot randomized placebo controlled phase II trial in acute ischemic stroke. Stroke..

[14] Orbegozo Cortés D, Su F, Santacruz C, Hosokawa K, Donadello K, Creteur J (2016). Ischemic conditioning protects the microcirculation, preserves organ function, and prolongs survival in sepsis. Shock..

[15] Joseph B, Khalil M, Hashmi A, Hecker L, Kulvatunyou N, Tang A (2017). Survival benefits of remote ischemic conditioning in sepsis. J Surg Res.

[16] Kim YH, Yoon DW, Kim JH, Lee JH, Lim CH (2014). Effect of remote ischemic post-conditioning on systemic inflammatory response and survival rate in lipopolysaccharide-induced systemic inflammation model. J Inflamm (Lond).

[17] Chan AW, Tetzlaff JM, Altman DG, Dickersin K, Moher D (2013). SPIRIT 2013: a new guidance for content of clinical trial protocols. Lancet..

[18] Vincent JL, Moreno R, Takala J, Willats S, De Mendonça A, Bruining H (1996). The SOFA (Sepsis-related Organ Failure Assessment) score to describe organ dysfunction/failure. Intensive Care Med.

[19] Ferreira FL, Bota DP, Bross A, Mélot C, Vincent JL (2001). Serial evaluation of the SOFA score to predict outcome in critically ill patients. JAMA..

[20] Argaud L, Cour M, Dubien PY, Giraud F, Jossan C, Riche B (2016). JAMA Cardiol.

[21] Cour M, Bresson D, Hernu R, Argaud L (2016). SOFA score to assess the severity of the post-cardiac arrest syndrome. Resuscitation..

[22] Singer M, Deutschman CS, Seymour CW, Shankar-Hari M, Annane D, Bauer M (2016). The Third International Consensus Definitions for Sepsis and Septic Shock (Sepsis-3). JAMA..

[23] Pickering TG, Hall JE, Appel LJ, Falkner BE, Graves J, Hill MN (2005). Recommendations for blood pressure measurement in humans and experimental animals: Part 1: Blood pressure measurement in humans: a statement for professionals from the Subcommittee of Professional and Public Education of the American Heart Association Council on High Blood Pressure Research. Hypertension..

[24] Brunkhorst FM, Oppert M, Marx G, Bloos F, Ludewig K, Putensen C (2012). Effect of empirical treatment with moxifloxin in meropenem vs meropenem on sepsis-related organ dysfunction in patients with severe sepsis: a randomized trial. JAMA..

[25] Gordon AC, Perkins GD, Singer M, McAuley DF, Orme RM (2016). Levosimensan for the prevention of acute organ dysfunction in sepsis. N Engl J Med.

[26] Schulz KF, Altman DG, Moher D, for the CONSORT Group (2010). CONSORT 2010 Statement: updated guidelines for reporting parallel group randomised trials. BMJ..

[27] Boutron I, Moher D, Altman D, Schulz K, Ravaud P, for the CONSORT Group. Methods and processes of the CONSORT Group (2008). Example of an extension for trials assessing nonpharmacologic treatments. Ann Intern Med.

